# The Impact of Sugar Beet Seed Pelletization on the Proliferation of Nematophagous Fungi

**DOI:** 10.3390/microorganisms13081936

**Published:** 2025-08-19

**Authors:** Miroslava Soukupová, David Novotný

**Affiliations:** Ecology, Diagnostics and Genetic Resources of Agriculturally Important Viruses, Fungi and Phytoplasmas, Division of Crop Protection and Plant Health, Czech Agrifood Research Center, Drnovská 507/73, Ruzyně, 161 00 Praha, Czech Republic; david.novotny@carc.cz

**Keywords:** nematophagous fungi, inhibition, pelletization seed, sugar beet

## Abstract

Pelleting seeds to enhance sowing conditions through the incorporation of pesticides or fertilizers has become a prevalent agricultural practice. This study sought to evaluate the effect of pelletized seeds and the substances they release in the form of an extract on four species of nematophagous fungi. The fungus *Pleurotus ostreatus* was the most sensitive to the presence of pelletized seeds; the growth of all three evaluated strains of *P. ostreatus* was inhibited from 42.84 to 94.33% compared to the control. In the case of the fungi *Stropharia rugosoannulata* and *Orbilia oligospora*, a statistically significant inhibition of the growth of all three evaluated strains was observed, though this inhibition was less pronounced than in the case of *P. ostreatus*. In contrast, the inhibitory effect on the fungus *Clonostachys rosea* exhibited a lower inhibition of mycelial growth (0.65–20.41%) compared to the control. The selection of suitable strains of nematophagous fungi tolerant to substances used for seed pelletization could assist in the management of nematodes. The inoculum of nematophagous fungi can be used in sugar beet sowing as a supplement, but fungi should not be used as part of the seed coatings because their viability is most inhibited in direct contact with fungicides.

## 1. Introduction

Sugar beet (*Beta vulgaris* ssp. *Vulgaris* L.) (Plantae: Magnoliophyta: Rosopsida: Caryophyllales: Amaranthaceae) is a significant economic crop, responsible for 20% of global sugar production [1]. In the majority of European and American production areas, seeds of sugar beet are pelleted. Pelletization involves the coating of seeds with a layer of inert material, thereby unifying the original shape and size of the seed, thus increasing its weight and improving its plantability. The most common pellet sizes range from 3.50 to 4.75 mm [2]. The primary function of pelletizing is to enhance the quality of sowing and seed distribution, particularly in precision sowing, and it also facilitates the direct application of chemicals, fertilizers, and growth agents to the seed. The layer contains various pesticides (mainly insecticides and fungicides). The substances thiamethoxam, tefluthrin, hymexazole, tetramethylthiuram disulphide, and bis(dimethylthiocarbamoyl)disulphide are most commonly used as seed treatment pesticides to protect sugar beet seed.

The beet cyst nematode (*Heterodera schachtii* Schmidt) (Animalia: Nematoda: Secernentea: Tylenchida: Heteroderidae) is considered to be one of the most economically significant pests affecting sugar beets. To mitigate the impact of this pest, a four-year cycle involving the incorporation of sugar beets and other host crops into the crop rotation, including the cultivation of special cash crops, has been implemented. Conventional chemical nematicides are being progressively phased out in favor of the utilization of nematophagous fungi, which serve as a more environmentally friendly biological control mechanism for plant parasitic nematodes [3].

Nematophagous fungi are distinguished by their capacity to capture or destroy nematodes, thereby exerting a regulatory effect on the population size of these parasites on plants. These fungi employ unique mycelial structures for the purpose of capturing nematode eggs and cysts. The manner in which these fungi infect nematodes is the basis of their classification into four primary groups: (a) nematode-trapping fungi, which produce extensive hyphal networks, knobs, and constricting rings as trapping devices to catch and hold live nematodes; (b) endoparasitic fungi, which exist as obligate parasites that exist as conidia in the environment and infect nematodes by either adhering to the surface of the prey or by directly being ingested by the nematodes, followed by germination, growth, and nematode killing; (c) egg- and cyst-parasitic fungi, as facultative parasites that grow on and parasitize the sedentary stages of nematodes such as eggs; and (d) toxin-producing fungi, producing toxic compounds that are active against nematodes [4].

The taxonomic classification of nematophagous fungi encompasses a diverse array of over 200 species, which are distributed across all major fungal groups, including oomycetes, chytridiomycetes, zygomycetes, ascomycetes, and basidiomycetes [5]. *Orbilia oligospora* (Fresen.) Baral & E. Weber (formerly *Arthrobotrys oligosporus* Fresen.) (Fungi: Ascomycota: Pezizomycotina: Orbiliomycetes: Orbiliomycetidae: Orbiliales: Arthrobotryaceae) is the most prevalent soil-dwelling fungus that uses chemotaxis to hunt nematodes [6]. It releases various substances into its environment, including attractive volatile compounds, antibiotics, and toxic substances, as well as toxic terpene compounds, which combine to form sticky substances in the traps it uses [7]. Upon contact with nematodes, the fungus undergoes a spontaneous differentiation of its mycelia into functional structures, which include traps that adhere to the nematodes and result in their demise [8]. The formation of the initial branch is initiated by a parent hypha that curves to connect with a pin formed on the parent hypha, thereby forming a loop composed of three cells. New branches formed from the initial loop can form new loops and their nets [9,10]. The *Stropharia rugosoannulata* Farl. ex Murrill (Fungi: Basidiomycota: Agaricomycotina: Agaricomycetes: Agaricomycetidae: Agaricales: Strophariaceae) is a lesser-known and less commonly cultivated edible mushroom [11]. This species is distributed in northern temperate areas. This fungus also forms trapping structures called acanthocytes, which are large star-shaped cells whose protrusions perforate and immobilize the nematode [12]. *Clonostachys rosea* (Link) Schroers, Samuels, Seifert & W. Gams (Fungi: Ascomycota: Pezizomycotina: Sordariomycetes: Hypocreomycetidae: Hypocreales: Bionectriaceae) is a fungal species that is commonly found in soil. It produces extracellular proteases that allow it to immobilize the nematode and hydrolyze cuticle proteins [13]. In addition to this, *C. rosea* is a very effective bioagent against a few fungal pathogens and insects [14,15,16]. *Pleurotus ostreatus* (Jacq.) P. Kumm. (Fungi: Basidiomycota: Agaricomycotina: Agaricomycetes: Agaricomycetidae: Agaricales: Pleurotaceae) is among the most cultivated fungi in the world. It is highly prized for its applications in the food, medicine, and biotechnology industries. The fungus forms so-called toxocysts [17], also known as sticky buttons [18] or sticky nodules [10]. These are a combination of toxic action and trapping structure—directed hyphae [18,19].

The inoculation of sugar beet fields with fungal nematophagous organisms has been demonstrated to enhance root weight, sugar content, and plant root quality [3,20]. However, the presence of nematophagous fungi in the soil can be negatively affected by residues of fungicides or other chemicals. The influence of pesticides on nematophagous fungi was investigated by Mensin et al. [21], Bengtsson S. [22], and Goltapeh et al. [23]. The objective of this study was to ascertain whether substances commonly utilized for pelleting sugar beet seeds, including fungicides, impact the growth of these fungi. The study evaluated the effect of pelleted seeds from several different manufacturers and their inhibitory effect on different strains of nematophagous fungi.

## 2. Materials and Methods

### 2.1. Microorganisms

The fungal strains utilized in the present study were sourced from two public culture collections of microorganisms: the Culture Collection of Microorganisms of the Czech Agrifood Research Center (VURV) (Prague, Czech Republic) and the Culture Collection of Fungi (CCF) (Department of Botany, Faculty of Science, Charles University, Prague, Czech Republic): *Orbilia oligospora* VURV-F 564, *O. oligospora* VURV-F 565, *O. oligospora* VURV-F 566, *Clonostachys rosea* VURV-F 156, *C. rosea* VURV-F 224, *C. rosea* CCF 3222, *Pleurotus ostreatus* VURV-F 5145, *P. ostreatus* VURV-F 5147, *P. ostreatus* VURV-F 5150, *Stropharia rugosoannulata* VURV-F 5083, *S. rugosoannulata* VURV-F 5084, *S. rugosoannulata* VURV-F 5132.

### 2.2. Seeds

The used seeds were produced by the following five producers: KWS SAAT SE & Co (Einbeck, Germany), Strube D&S GmbH (Söllingen, Germany), SESVanderHave (Tienen, Belgium), Syngenta Crop Protection AG (Basel, Switzerland), and MARIBO DLF Beet Seed ApS (Holeby, Denmark). In the ensuing text, the abbreviations of the manufacturers’ names will be employed, namely KWS, Stube, Sesvander, Syngenta, and Maribo Seed, respectively.

The seeds were all subjected to the same insecticide and fungicide treatment. The insecticide, known commercially as Cruiser Force, is formulated to target sucking and feeding pests and contains the active substance thiamethoxam, which belongs to the neonicotinoid group, at a concentration of 60 g/SU (SU denotes a sowing unit of 100,000 seeds per hectare). The formulation also incorporates the active substance tefluthrin, which belongs to the pyrethroid group [24]. The fungicide Tachigaren 70 WP, containing 70% of the active substance hymexazol, is employed in the management of black leg of beet [25], and the fungicide Thiram 42-S with 42% of the active substance thiram [26]. The pelleted seeds differed only in the use of the coating agent, its quantity, and the amount of micro- and macro-elements added for seed nutrition. All seeds used in this study were provided by the Beet Institute (Řepařský institut spol. s r. o., Semčice, Czech Republic) and their producers had no interest in this study.

### 2.3. Methods

In the first part of the study, the influence of the pelleted seed on the growth of fungal mycelia was evaluated. An unsterilized seed was positioned at a distance of 1 cm from the fungus on a 2% malt agar (malt extract—VWR International, Leuven, Belgium; agar—Roth, Karlsruhe, Germany) in 90 mm Petri dish.

In the second part of the experiment, a suspension of the envelope material in water was prepared from the pelleted seeds. This was performed to simulate the gradual leaching of active ingredients in the environment due to soil moisture or rainwater. A single seed was shaken in 2, 8, and 32 mL of sterile distilled water at 700 rpm for a duration of 15 min. The resulting suspensions were sterilized using syringe filters with a pore size of 0.2 nm. Thereafter, 300 µL of the aforementioned suspension was applied to a Petri dish containing 2% malt agar (malt extract—VWR International, Leuven; agar—Roth, Karlsruhe), and the suspension was spread with a Drigalski spatula over the entire surface of the agar plate. An equal volume of distilled sterile water was utilized as a control. Following the drying process, a 1 mm diameter mycelial target of the fungi under evaluation was inoculated into the center of the agar plate. Six replicates were performed for each variant. The Petri dishes were then placed in conditions of darkness at a temperature of 25 °C for a seven-day cultivation period. Following this, the colony size formed on the Petri dishes was measured, with two perpendicular measurements for each Petri dish. The results obtained in experimental variants were compared with the control. Inhibition rate of fungal growth was then calculated according to the following equation [27]:

$$\mathrm{Inhibition}\;=\;\frac{\mathrm{Colony}\;\mathrm{size}\;\mathrm{on}\;\mathrm{control}\;\mathrm{plate}-\mathrm{Collony}\;\mathrm{size}\;\mathrm{on}\;\mathrm{fungicide}\;\mathrm{plate}-\mathrm{inoculum}\;\mathrm{size}}{\mathrm{Colony}\;\mathrm{size}\;\mathrm{on}\;\mathrm{control}\;\mathrm{plate}-\mathrm{inoculum}\;\mathrm{size}}\;\times \;100\%$$

The obtained data were evaluated by analysis of variance (ANOVA) and Fisher’s least significant difference (LSD) method in TIBCO Statistica Ultimate Academic software (version 13.5; StatSoft (Europe) GmbH, Hamburg, Germany).

## 3. Results

**Figure 1:**
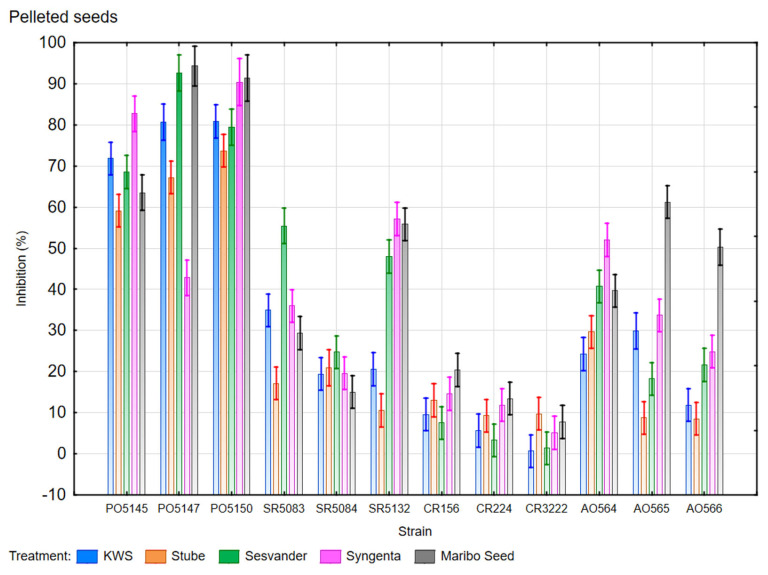
Growth inhibition in percent of fungi *P. ostreatus* (marked as PO strain: VURV-F 5145, VURV-F 5147, VURV-F 5150), *S. rugosoannulata* (marked as SR strain: VURV-F 5083, VURV-F 5084, CPPF 5132), *C. rosea* (marked as CR strain: VURV-F 156, VURV-F 224, CCF 3222), and *O. oligospora* (marked as AO strain: VURV-F 564, VURV-F 565, VURV-F 566) caused by the presence of pelleted seeds from five different manufacturers (treatment). Control is 0%. ANOVA with Fisher’s LSD post hoc test (α = 0.05; *n* = 12); the error bar is the standard deviation (unweighted mean).

In the case of evaluating the effect of the presence of pelleted sugar beet seeds on the growth of the evaluated fungal strains, it can be stated that the presence of all pelleted seeds from all five producers inhibited the growth of all evaluated nematophagous fungal strains, as shown in Figure 1.

The evaluation carried out on Petri dishes showed that the fungus *P. ostreatus* was the most sensitive to the presence of pelletized seeds of all four fungal species studied. In the presence of pelletized seeds at a distance of 1 cm from the inoculated fungus, the growth of all three evaluated strains of *P. ostreatus* was inhibited by more than 50% (from 59.14% to 94.33%) compared to the growth of these fungal strains in the control, except for strain VÚRV-F 5147, which, in the presence of seeds from Syngenta, showed a lower inhibition of 42.84%.

In the case of the evaluation of the *S. rugosoannulata*, a statistically significant inhibition of the growth of all three evaluated strains was observed, although this inhibition was smaller than that observed in the case of the fungus *P. ostreatus*. The inhibition of the growth of the fungus *S. rugosoannulata* ranged from 10.58% to 57.17%. As can be seen in graph 1, this large variance is due to large differences in the sensitivity of strains VURV-F 5083 and VURV-F 5132 to different pelleted seeds. For example, strain VURV-F 5083 showed 55.45% inhibition when it was cultivated with seed from Sesvander, but with seeds from other companies the inhibition was below 40%, and with seed from Stube, inhibition was only 17.15%. This difference could be caused by different rates of fungicide release from different seeds due to their hardness or by some other substance present in the pellet that may have differed between seeds from different manufacturers.

Similarly, the inhibitory effect on the fungus *O. oligospora* exhibited variation among the evaluated strains, and the origin of the pelletized seed also had a significant impact. The growth of the VURV-F 564 strain was statistically significantly inhibited (from 24.26% to 52.01%) in the presence of all five evaluated seed variants. The seed from Stube affected the growth of VURV-F 565 and VURV-F 566 strains to a small extent, and its effect was statistically insignificant. However, the effect on *O. oligospora* was found to be statistically significant for the remaining four seeds from different manufacturers, with the highest recorded inhibition being observed in seeds from Maribo Seed, which inhibited the growth of strain VURV-F 565 by 61.22% and strain VURV-F 566 by 50.30%.

Growth inhibition of *C. rosea* in the presence of pelleted seeds was the least significant compared to the other fungal species evaluated. There was little difference between the strains examined, and the growth of strain CCF 3222 was statistically insignificantly suppressed. All seed variants inhibited the growth of all *C. rosea* strains within 0.65–20.41%.

The seed tests were supplemented by an evaluation of the effect of seed coat leachate at 2, 18, and 32 milliliters of distilled water. This evaluation was intended to simulate the effect of substances leached from the seed coat by rain or irrigation on nematophagous fungi. These fungi would not be in direct contact with the pelleted seed but would be located in deeper soil layers or at a greater distance from the sown seeds.

**Figure 2:**
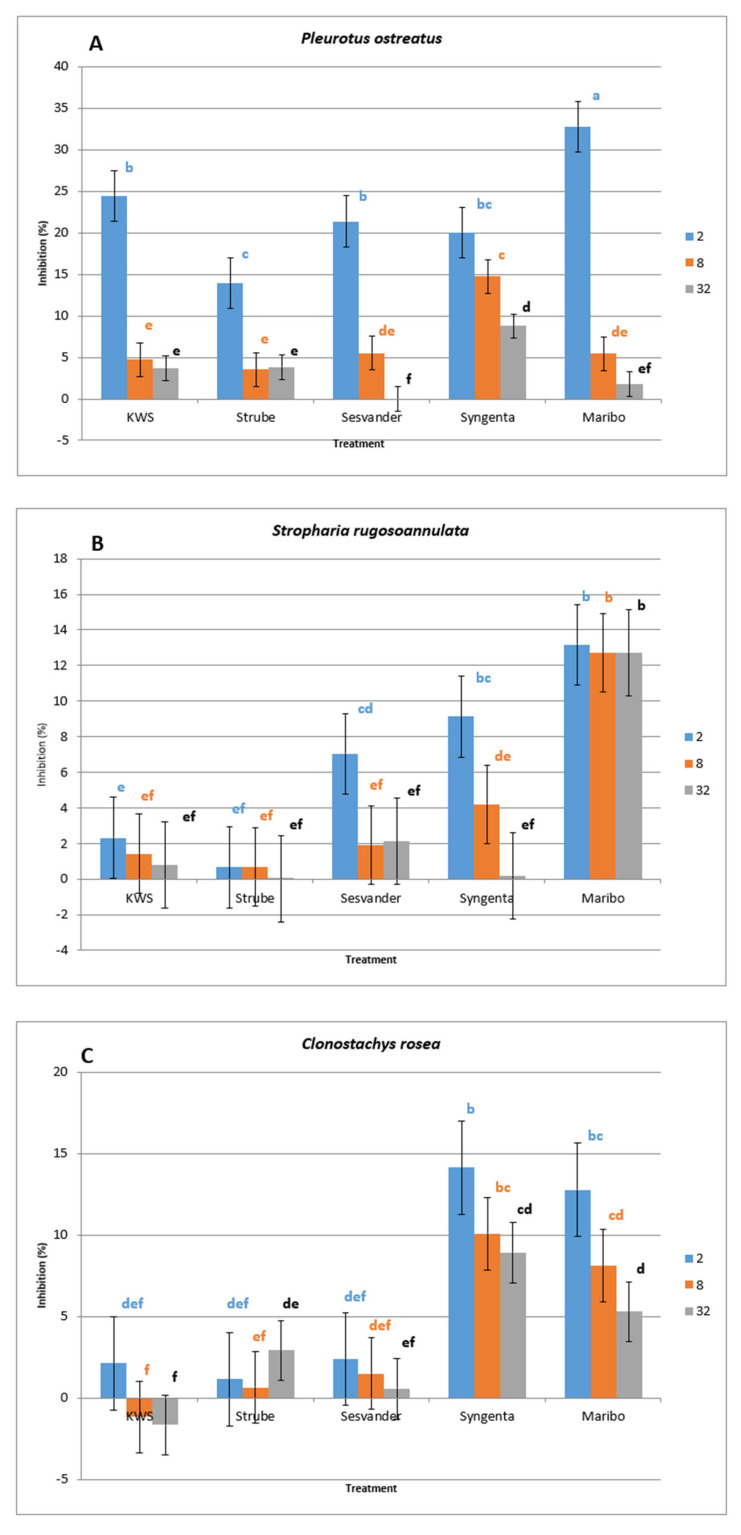

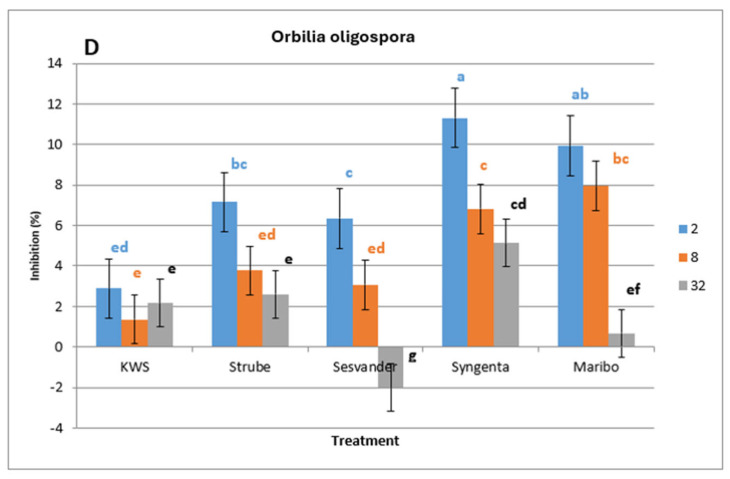
Growth inhibition (in percent) of investigated fungal species (**A**) *P. ostreatus*, (**B**) *S. rugosoannulata*, (**C**) *C. rosea,* and (**D**) *O. oligospora* caused by the presence of seed coat leachate from five different manufacturers and three concentrations of the coat leachate. Treatments 2, 8, and 32 mean dilutions at 2, 8, and 32 milliliters of distilled water. The error bar is standard deviation. Significantly different classes of values are indicated in the form of letters; control is 0%, and the class with the letter f.

The evaluation of the effect of seed coat leachate on nematophagous fungi indicates that leaching with a higher extract seed coat content exerts a higher inhibitory effect usually and, in some cases, a statistically significant stimulatory effect on the growth of the evaluated strains of nematophagous fungi. As demonstrated in Figure 2, in general, it can be stated that for all evaluated nematophagous fungi, the degree of inhibition was influenced by the dilution of the extract, which led to a slight stimulation of fungal growth compared to the control. In most cases, however, the values of the inhibition or stimulation of the mycelial growth of the studied nematophagous fungi on agar with seed extracts were statistically insignificant compared to the control on plates treated with distilled water only.

As was the case with pure pelleted seeds, the most sensitive fungal species in the case of seed extracts was *Pleurotus ostreatus* (inhibition rate 13.94–32.76%). All strains of this fungus exhibited significant inhibition in the presence of seed extract in 2 mL of distilled water. In contrast, more diluted extracts exerted a lesser inhibitory effect. At the highest extract concentration, the inhibition of *P. ostreatus* was observed to be the highest in the seed coat leachate from Maribo and KWS. The lowest level of inhibition was found when cultured on agar medium with Strube seed coat leachate.

At the highest concentration of extract, the growth inhibition rate of *S. rugosoannulata* ranged from 2.32 to 13.17% and was highest when cultured on medium with Maribo seed extract. Lower concentrations of Maribo seed extract also visibly inhibited the growth of this fungal species. The lowest level of inhibition was found when cultured on agar medium with Strube and KWS seed coat leachate.

Seed coat extracts from Syngenta and Maribo inhibited *C. rosea* growth more than KWS, Strube, and Sesvander extracts. At the highest concentration of the extract, the growth inhibition rate of *C. rosea* ranged from 2.11 to 14.13%. Lower concentrations of Maribo and Syngenta seed extract also visibly inhibited the growth of this fungus. At lower concentrations of KWS seed coat extract, this fungus species grew more compared to the control.

At the highest extract concentration, the growth inhibition rate of *O. oligospora* ranged from 2.92 to 11.31% and was highest when cultured on media with Syngenta and Maribo seed extracts. The lowest level of inhibition at the highest concentration of extract was found when *O. oligospora* was cultured on agar medium with KWS seed coat leachate.

Maribo seed coat extract inhibited the growth of all the fungi studied the most compared to other seed coat extracts. The second most inhibitory extract for most fungal species was the Syngenta seed coat extract. In contrast, KWS seed coat extract, except for *P. ostreatus*, suppressed the growth of all fungal species the least.

## 4. Discussion

Pelleting seeds to enhance sowing conditions through the incorporation of pesticides or fertilizers has become a prevalent agricultural practice in contemporary times [28]. The majority of existing studies have focused on the impact of these substances on cultivated crops [29,30]. However, there is a paucity of studies addressing the consequences of the substances released from pelletized seeds on the microbial composition of soil. Soil contains a diverse microbiota, comprising both pathogenic microorganisms that are targeted by the application of fungicides to crop seeds [31] and microorganisms that are beneficial to crops [32,33], including nematophagous fungi [34]. This study aimed to contribute to the existing body of knowledge by comparing the effect of pelletized seeds and the substances they release in the form of an extract on four species of nematophagous fungi.

The evaluation of the study revealed that the fungus *P. ostreatus* was the most sensitive to the presence of pelletized seeds. The effect of the fungicidal substances thiram and hymexazol on *P. ostreatus* is not yet fully known; however, other fungicides, such as prochloraz against fungal mycoparasites, are commonly utilized in oyster mushroom production [35]. Consequently, it was hypothesized that *P. ostreatus* would demonstrate a reduced sensitivity to the substances contained in pelleted seeds. However, the findings of this study demonstrated an opposite response, with *P. ostreatus* being identified as the fungus on which tested pelletized seeds had the most significant inhibitory effect. A similar outcome was observed for *S. rugosoannulata*, where the inhibitory effect was lower than for *P. ostreatus*, yet it was statistically significant for all three strains. This species of mushroom is also cultivated for consumption, though it is not as widely cultivated as the oyster mushroom [36]. The inhibitory effect of pelletized seeds on *O. oligospora* was observed to vary between 8.51% and 61.22%, contingent on the fungal strain and the origin of the seeds. Kumar et al. [37] also observed that *A. dactyloides* demonstrated a heightened sensitivity to thiram, with complete inhibition of conidial trap formation and conidium germination at concentrations of 20 mg/kg and above. In the present study, all three evaluated strains of *C. rosea* exhibited the lowest level of inhibition against the pelleted seeds and substances contained in their coatings that were tested. Similarly, Roberti et al. [38] reported that the strain of *C. rosea* that was examined was the least sensitive to thiram regarding radial mycelial growth and conidia germination from all tested antagonists. A small negative-to-positive effect of these leachates on fungal growth was observed at lower concentrations of the suspension leached from pesticide-treated pelleted seed. This may be due to the presence of substances that fungi can use for their nutrition [2]. A similar effect can be assumed in vivo (in soil), but it would be advisable to verify this through further research, as in a system that includes soil, the plant, seed coating materials, the consortium of microorganisms present in the soil, and any added inoculum of nematophagous fungi, our findings will be influenced by a large number of interactions between these biotic and abiotic factors. All studies to date have only looked at the effect on plant yield [29,39], not on the ecosystem into which the coated seeds are introduced. Some studies evaluate the effect of seed pelleting as protection against abiotic stress, e.g., moisture [40], salinity [41], or acidity [42], but none have yet addressed the effect of pelleted seeds on the soil microbiome. Seed pelleting is widely used today not only in agricultural practice [2,43] but also for seeds of native vegetation species in the restoration of terrestrial ecosystems [44,45], but even in this case, only the effect on the sown plants was evaluated, not the effect of substances released from the pellets on the microbiome in the given location.

Plant microbial communities are dynamic systems that change over time [46], and the seed germination phase is one of the most vulnerable stages of plant development, during which the plant can be positively or negatively affected by various abiotic or biotic factors [47]. Also, communities are influenced by biotic (including cultivated plants themselves) and abiotic factors. Endophytic microorganisms in seeds are one source of root community microbiota (including the rhizosphere and endosphere of roots). Soil microbial communities are another source of organisms that can become a part of the root microbial community. The genotype of the plant also influences the composition of microbial communities. Cultivar-specific differences in seed and root microbial communities have been reported [48]. The C/N ratio is another factor that influences the composition of the root microbiome and the soil around the roots [49]. The composition of pelleted seeds can also influence the composition of the root microbiota. It has been found that pesticides contained in pelleted seeds positively influence plant germination rates and plant biomass when there is a higher concentration of fungi in the soil [50]. Organisms used in biological crop protection (including nematophagous fungi) can also influence this microbial community. The four fungal species studied have different ecologies and therefore can be expected to have different relationships with microorganisms associated with beet roots. *C. rosea* is a common soil fungus, colonizing mainly decaying plants, with a wide distribution and a globally distributed biocontrol agent [51]. Its influence on the microbiota of beet roots and germinating plants and the soil around these plants will be greater than that of *P. ostreatus* and *S. rugosoannulata* (both growing on substrates with higher concentrations and larger amounts of cellulose) or that of *O. oligospora*, a well-known nematode-hunting fungus found in various substrates, including soil and the rhizosphere of plants [51]. Further research would be needed to determine their impact on the mycobiota of beets.

The method of application to the field of the studied fungal species depends on their ability to produce spores when cultivated in vitro or semi-in vitro conditions. Species (*C. rosea*, partially *O. oligospora*) that often produce spores during in vitro cultivation can be added in the form of a spore suspension to the seed when sowing beets. *P. ostreatus* and *S. rugosoannulata* do not produce spores under in vitro or semi-in vitro conditions and can be incorporated into the soil in the form of mycelium when preparing the field for beet cultivation before sowing. Beneficial nematodes used in biological crop protection can be attacked by the nematophagous fungi under investigation, so it makes no sense to apply them simultaneously.

## 5. Conclusions

This study set out to evaluate the effect of pelletized seeds and the substances they release in the form of an extract on four species of nematophagous fungi. The evaluation of the study revealed that the fungus *P. ostreatus* was the most sensitive to the presence of pelletized seeds. In the case of the fungi *S. rugosoannulata* and *O. oligospora*, a statistically significant inhibition of the growth of all three strains evaluated was observed, although this inhibition was less pronounced than in the case of the fungus *P. ostreatus*. Conversely, the inhibitory effect on the fungus *C. rosea* exhibited a lower inhibition of mycelial growth (0.65–9.74%) compared to the control. The highest inhibitory effect was observed when the fungal culture was in direct contact with the pesticide-treated seed, and lower concentrations of the suspension leached from the pesticide-treated pelleted seed caused a small negative-to-positive effect on the growth of the fungi studied. Suspensions leached from pesticide-treated pelleted seed do not completely inhibit or significantly suppress the growth of the studied nematophagous fungi. This study showed that inoculum of nematophagous fungi can be used in sugar beet sowing as a supplement in combination with pesticide-containing mordants. The selection of suitable strains of nematophagous fungi tolerant to substances used for seed pelletization could assist in the management of nematodes. This approach could lead to the promotion of higher microbial diversity and the sustainability of agricultural soils. However, fungi should not be used as part of the seed coatings because their viability is most inhibited in direct contact with fungicides, and they may not survive the seed pelletization process.

## Data Availability

The original contributions presented in this study are included in the article. Further inquiries can be directed to the corresponding author. Basic data will be made available on request.
